# Supplementary material for: The Gut Bacteria Dysbiosis Contributes to Chronic Graft-Versus-Host Disease Associated With a Treg/Th1 Ratio Imbalance
Source: Front Microbiol. 2022 Sep 8;13:813576. doi: 10.3389/fmicb.2022.813576 (PMC9493085; doi:10.3389/fmicb.2022.813576)
Supplement: Supplementary file 1 [file Data_Sheet_1.zip › P101SC18090073-01-B1-3-4_result/05.WebShow/taxa_charts_group_html/charts/hU9AEhM1ZyY4d9KGEZPMlbbTceRXtk_legend.pdf]

k__Archaea;p__Euryarchaeota;c__Methanobacteria;o__Methanobacteriales;f__Methanobacteriaceae
k__Bacteria;Other;Other;Other;Other
k__Bacteria;p__Acidobacteria;c__Holophagae;o__;f__
k__Bacteria;p__Acidobacteria;c__unidentified_Acidobacteria;o__unidentified_Acidobacteria;f__Pyrinomonadaceae
k__Bacteria;p__Acidobacteria;c__unidentified_Acidobacteria;o__unidentified_Acidobacteria;f__unidentified_Acidobacteria
k__Bacteria;p__Actinobacteria;c__Acidimicrobiia;Other;Other
k__Bacteria;p__Actinobacteria;c__Acidimicrobiia;o__;f__
k__Bacteria;p__Actinobacteria;c__Coriobacteriia;o__Coriobacteriales;f__Atopobiaceae
k__Bacteria;p__Actinobacteria;c__Rubrobacteria;o__Rubrobacterales;f__unidentified_Rubrobacterales
k__Bacteria;p__Actinobacteria;c__Thermoleophilia;o__Gaiellales;f__
k__Bacteria;p__Actinobacteria;c__Thermoleophilia;o__Solirubrobacterales;Other
k__Bacteria;p__Actinobacteria;c__Thermoleophilia;o__Solirubrobacterales;f__
k__Bacteria;p__Actinobacteria;c__unidentified_Actinobacteria;o__Actinomycetales;f__Actinomycetaceae
k__Bacteria;p__Actinobacteria;c__unidentified_Actinobacteria;o__Bifidobacteriales;f__Bifidobacteriaceae
k__Bacteria;p__Actinobacteria;c__unidentified_Actinobacteria;o__Corynebacteriales;f__Corynebacteriaceae
k__Bacteria;p__Actinobacteria;c__unidentified_Actinobacteria;o__Corynebacteriales;f__Nocardiaceae
k__Bacteria;p__Actinobacteria;c__unidentified_Actinobacteria;o__Micrococcales;f__Microbacteriaceae
k__Bacteria;p__Actinobacteria;c__unidentified_Actinobacteria;o__Propionibacteriales;f__Nocardioidaceae
k__Bacteria;p__Armatimonadetes;c__Fimbriimonadia;o__Fimbriimonadales;f__Fimbriimonadaceae
k__Bacteria;p__Bacteroidetes;c__Bacteroidia;o__Bacteroidales;f__Bacteroidaceae
k__Bacteria;p__Bacteroidetes;c__Bacteroidia;o__Bacteroidales;f__Barnesiellaceae
k__Bacteria;p__Bacteroidetes;c__Bacteroidia;o__Bacteroidales;f__Marinifilaceae
k__Bacteria;p__Bacteroidetes;c__Bacteroidia;o__Bacteroidales;f__Muribaculaceae
k__Bacteria;p__Bacteroidetes;c__Bacteroidia;o__Bacteroidales;f__Porphyromonadaceae
k__Bacteria;p__Bacteroidetes;c__Bacteroidia;o__Bacteroidales;f__Prevotellaceae
k__Bacteria;p__Bacteroidetes;c__Bacteroidia;o__Bacteroidales;f__Rikenellaceae
k__Bacteria;p__Bacteroidetes;c__Bacteroidia;o__Bacteroidales;f__Tannerellaceae
k__Bacteria;p__Bacteroidetes;c__Bacteroidia;o__Chitinophagales;f__Chitinophagaceae
k__Bacteria;p__Bacteroidetes;c__Bacteroidia;o__Cytophagales;Other
k__Bacteria;p__Bacteroidetes;c__Bacteroidia;o__Flavobacteriales;f__Flavobacteriaceae
k__Bacteria;p__Bacteroidetes;c__Bacteroidia;o__Flavobacteriales;f__unidentified_Flavobacteriales
k__Bacteria;p__Bacteroidetes;c__Bacteroidia;o__Sphingobacteriales;f__
k__Bacteria;p__Deinococcus-Thermus;c__Deinococci;o__Deinococcales;f__Deinococcaceae
k__Bacteria;p__Firmicutes;Other;Other;Other
k__Bacteria;p__Firmicutes;c__Bacilli;o__Bacillales;f__Staphylococcaceae
k__Bacteria;p__Firmicutes;c__Bacilli;o__Bacillales;f__unidentified_Bacillales
k__Bacteria;p__Firmicutes;c__Bacilli;o__Lactobacillales;f__Enterococcaceae
k__Bacteria;p__Firmicutes;c__Bacilli;o__Lactobacillales;f__Lactobacillaceae
k__Bacteria;p__Firmicutes;c__Bacilli;o__Lactobacillales;f__Leuconostocaceae
k__Bacteria;p__Firmicutes;c__Bacilli;o__Lactobacillales;f__Streptococcaceae
k__Bacteria;p__Firmicutes;c__Clostridia;o__Clostridiales;Other
k__Bacteria;p__Firmicutes;c__Clostridia;o__Clostridiales;f__Christensenellaceae
k__Bacteria;p__Firmicutes;c__Clostridia;o__Clostridiales;f__Eubacteriaceae
k__Bacteria;p__Firmicutes;c__Clostridia;o__Clostridiales;f__Lachnospiraceae
k__Bacteria;p__Firmicutes;c__Clostridia;o__Clostridiales;f__Peptococcaceae
k__Bacteria;p__Firmicutes;c__Clostridia;o__Clostridiales;f__Peptostreptococcaceae
k__Bacteria;p__Firmicutes;c__Clostridia;o__Clostridiales;f__Ruminococcaceae
k__Bacteria;p__Firmicutes;c__Clostridia;o__Clostridiales;f__unidentified_Clostridiales
k__Bacteria;p__Firmicutes;c__Erysipelotrichia;o__Erysipelotrichales;f__Erysipelotrichaceae
k__Bacteria;p__Firmicutes;c__Negativicutes;o__Selenomonadales;f__Acidaminococcaceae
k__Bacteria;p__Firmicutes;c__Negativicutes;o__Selenomonadales;f__Veillonellaceae
k__Bacteria;p__Fusobacteria;c__Fusobacteriia;o__Fusobacteriales;f__Fusobacteriaceae
k__Bacteria;p__Melainabacteria;Other;Other;Other
k__Bacteria;p__Oxyphotobacteria;c__unidentified_Oxyphotobacteria;o__unidentified_Oxyphotobacteria;f__unidentified_Oxyphotobacteria
k__Bacteria;p__Proteobacteria;Other;Other;Other
k__Bacteria;p__Proteobacteria;c__Alphaproteobacteria;o__Caulobacterales;f__Caulobacteraceae
k__Bacteria;p__Proteobacteria;c__Alphaproteobacteria;o__Rhizobiales;Other
k__Bacteria;p__Proteobacteria;c__Alphaproteobacteria;o__Rhizobiales;f__Beijerinckiaceae
k__Bacteria;p__Proteobacteria;c__Alphaproteobacteria;o__Rhizobiales;f__Rhizobiaceae
k__Bacteria;p__Proteobacteria;c__Alphaproteobacteria;o__Rhizobiales;f__Xanthobacteraceae
k__Bacteria;p__Proteobacteria;c__Alphaproteobacteria;o__Rhodospirillales;f__unidentified_Rhodospirillales
k__Bacteria;p__Proteobacteria;c__Alphaproteobacteria;o__Sphingomonadales;f__Sphingomonadaceae
k__Bacteria;p__Proteobacteria;c__Alphaproteobacteria;o__unidentified_Alphaproteobacteria;f__unidentified_Alphaproteobacteria
k__Bacteria;p__Proteobacteria;c__Deltaproteobacteria;o__Desulfovibrionales;f__Desulfovibrionaceae
k__Bacteria;p__Proteobacteria;c__Gammaproteobacteria;o__Alteromonadales;f__Shewanellaceae
k__Bacteria;p__Proteobacteria;c__Gammaproteobacteria;o__Enterobacteriales;f__Enterobacteriaceae
k__Bacteria;p__Proteobacteria;c__Gammaproteobacteria;o__Pasteurellales;f__Pasteurellaceae
k__Bacteria;p__Proteobacteria;c__Gammaproteobacteria;o__Pseudomonadales;f__Moraxellaceae
k__Bacteria;p__Proteobacteria;c__Gammaproteobacteria;o__Pseudomonadales;f__Pseudomonadaceae
k__Bacteria;p__Proteobacteria;c__Gammaproteobacteria;o__Xanthomonadales;f__Rhodanobacteraceae
k__Bacteria;p__Proteobacteria;c__Gammaproteobacteria;o__Xanthomonadales;f__Xanthomonadaceae
k__Bacteria;p__Proteobacteria;c__Gammaproteobacteria;o__unidentified_Gammaproteobacteria;f__Burkholderiaceae
k__Bacteria;p__Proteobacteria;c__Gammaproteobacteria;o__unidentified_Gammaproteobacteria;f__Neisseriaceae
k__Bacteria;p__Tenericutes;c__Mollicutes;o__;f__
k__Bacteria;p__Tenericutes;c__Mollicutes;o__Anaeroplasmatales;f__Anaeroplasmataceae
k__Bacteria;p__Verrucomicrobia;c__Verrucomicrobiae;o__Chthoniobacterales;f__Chthoniobacteraceae
k__Bacteria;p__Verrucomicrobia;c__Verrucomicrobiae;o__Verrucomicrobiales;f__Akkermansiaceae
k__Bacteria;p__unidentified_Bacteria;c__unidentified_Bacteria;o__Campylobacterales;f__Campylobacteraceae
k__Bacteria;p__unidentified_Bacteria;c__unidentified_Bacteria;o__Campylobacterales;f__unidentified_Campylobacterales
k__Bacteria;p__unidentified_Bacteria;c__unidentified_Bacteria;o__unidentified_Bacteria;f__unidentified_Bacteria
